# Using basic technology to screen for diabetic retinopathy in Fiji

**Published:** 2008-06

**Authors:** Sandeep Nakhate, Maria Walker, Jonathan Walker

**Affiliations:** Head of Ophthalmology Unit, Labasa Hospital, Labasa, Fiji.; Allen County Retinal Surgeons.; Allen County Retinal Surgeons, 7900 West Jefferson #300, Fort Wayne, IN 46804, USA. Email: jonwalker22@gmail.com

The World Health Organization (WHO) estimates that almost 12 per cent of Fijians have diabetes. Although there are no official figures on the prevalence of diabetic retinopathy, a complication of diabetes, it is the second most common cause of vision loss after cataract in our hospital in Labasa, Fiji.

One of the main problems with treating diabetic retinopathy is that patients remain asymptomatic until their disease is very advanced. They then present for evaluation when it may be too late to preserve their vision.

Ideally, we would like to identify people with diabetic retinopathy as early as possible so treatment can begin when it is more effective. However, screening large numbers of diabetic patients in clinics is difficult both because of the distances patients have to travel and because of limited health care resources (including human resources). In addition, people with diabetes are reluctant to make use of the health care system until their disease is very advanced. This is true both in Fiji and in developing countries around the world.

In order to provide a solution to this problem, we devised a simple photographic system to screen for diabetic retinopathy using a portable camera. This system could be implemented in conditions where there were no personnel experienced in fundus photography and where there was no financial support or information technology infrastructure. We decided to test this screening system in the field.

We chose a Topcon NW100 non-mydriatic camera (see picture above), because it was durable and easy to use (indeed, after practising with this camera for about an hour, a motivated student will be able to take acceptable fundus photographs). Although the camera can be used without mydriatics, it is much easier to photograph the retina if the pupils are dilated with 0.5 per cent (or 1 per cent) tropicamide, especially if there are no darkened rooms in which to take the photographs. One of the nurses at our hospital in Labasa was trained in the use of the camera; she had no previous experience with either ophthalmic photography or fundus examination.

**Figure:**
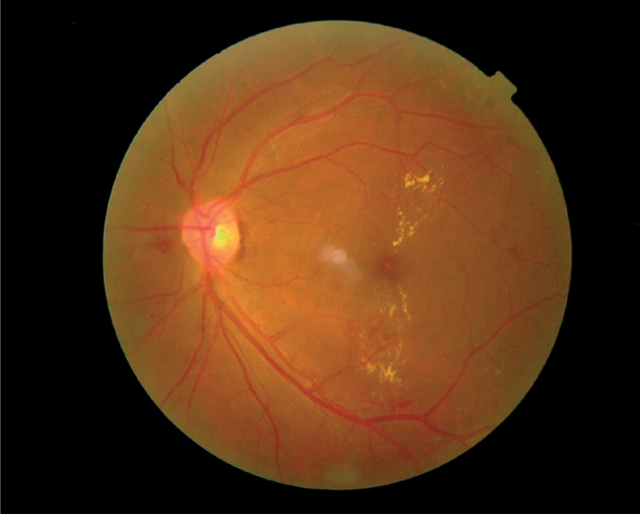
**Clinically significant macular oedema with hard exudates encroaching on the fovea, identified using photographic screening in an asymptomatic patient.**

**Figure:**
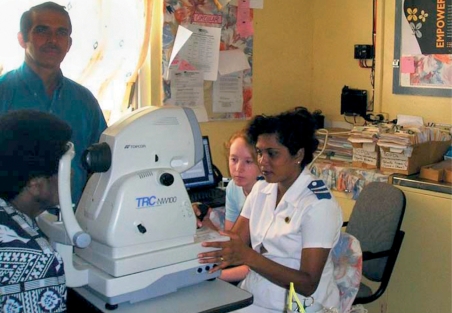
**The camera being used in an outlying medical clinic. FIJI**

This nurse travelled with the camera to small outlying medical clinics. There, she performed a preliminary examination with a torch to eliminate patients with obvious anterior segment problems, such as dense cataract, that would preclude photography. A total of 115 patients were photographed during this first screening mission. The images were stored on a laptop computer. An ophthalmologist at our hospital later analysed these images and provided a photographic diagnosis for each image.

In total, 75 per cent of the images (86 patients) were readable, meaning they were of sufficient quality to determine whether further evaluation was warranted. Nine patients showed signs of diabetic retinopathy which would require laser treatment. These patients were then contacted by the outlying clinics and arrangements were made to bring them to our hospital for evaluation. All of these patients came in for treatment and in all cases the photographic diagnosis was correct. In the remaining 25 per cent of cases, the images weren't readable; these patients were also encouraged to come to our hospital for a further examination.

We have since organised six further screenings. Of the 370 patients examined, a total of 30 per cent were found to have some degree of diabetic retinopathy and 8–9 per cent had retinopathy that was severe enough to require laser treatment. So far, all patients requiring laser have attended our hospital for treatment. The quality of the photographs has also improved with experience; far fewer pictures are now unreadable.

With this photographic screening technique, it is also possible to identify patients with milder degrees of retinopathy, who do not yet require treatment. This pool of patients is perhaps the most important, because they (and their physicians) can be made aware of any changes in their diabetes and of the need for improved control and monitoring of the disease.

Perhaps the most precarious aspect of this project is that everything depends on the ruggedness of the camera; we are very careful with maintaining and transporting it (no patients can be seen if the camera breaks). For instance, on the first day, the camera had been stored in an air-conditioned room; when it was taken outside it became soaked with condensation inside and out. Fortunately, it worked perfectly once it had dried out.

This screening method allows physicians' time to be used more efficiently, as they don't have to examine diabetic patients without retinopathy and can focus their attention on those needing treatment. This approach also has the advantage of identifying disease at an earlier stage, when treatment is both more effective and less time consuming.

